# Cerebral versus Ocular Visual Impairment: The Impact on Developmental Neuroplasticity

**DOI:** 10.3389/fpsyg.2016.01958

**Published:** 2016-12-26

**Authors:** Maria B. C. Martín, Alejandro Santos-Lozano, Juan Martín-Hernández, Alberto López-Miguel, Miguel Maldonado, Carlos Baladrón, Corinna M. Bauer, Lotfi B. Merabet

**Affiliations:** ^1^GIDFYS, European University Miguel de CervantesValladolid, Spain; ^2^Research Institute of Hospital 12 de Octubre (i+12)Madrid, Spain; ^3^Refractive Surgery and Visual Rehabilitation, Ophthalmology, Instituto Universitario de Oftalmobiología Aplicada, Eye InstituteValladolid, Spain; ^4^Laboratory for Visual Neuroplasticity, Massachusetts Eye and Ear Infirmary, Harvard Medical SchoolBoston, MA, USA

**Keywords:** cortical, cerebral, visual impairment, ocular blindness, connectivity

## Abstract

Cortical/cerebral visual impairment (CVI) is clinically defined as significant visual dysfunction caused by injury to visual pathways and structures occurring during early perinatal development. Depending on the location and extent of damage, children with CVI often present with a myriad of visual deficits including decreased visual acuity and impaired visual field function. Most striking, however, are impairments in visual processing and attention which have a significant impact on learning, development, and independence. Within the educational arena, current evidence suggests that strategies designed for individuals with ocular visual impairment are not effective in the case of CVI. We propose that this variance may be related to differences in compensatory neuroplasticity related to the type of visual impairment, as well as underlying alterations in brain structural connectivity. We discuss the etiology and nature of visual impairments related to CVI, and how advanced neuroimaging techniques (i.e., diffusion-based imaging) may help uncover differences between ocular and cerebral causes of visual dysfunction. Revealing these differences may help in developing future strategies for the education and rehabilitation of individuals living with visual impairment.

## Introduction

### The Nature of Visual Impairment and the Case of CVI

Humans are highly dependent on their sense of vision in order to interact with the surrounding world. Not surprisingly, the loss of visual function associated with blindness and visual impairment has a dramatic impact on an individual’s quality of life and independence (Dagnelie, 2013). Vision is a complex sensory function that requires the hierarchical participation of receptors (the photoreceptors of the eye), transmission (the optic nerves and optic radiations), and processing (the visual cortex) structures to transform captured visual information into meaningful percepts. Damage anywhere along the visual pathway typically results in some degree of visual impairment with characteristic clinical and functional manifestations. Traditionally, much of our understanding of visual impairment has been focused on the consequences of diseases and conditions affecting the eye and optic nerve (e.g., cataracts, macular degeneration, and glaucoma). Damage to these structures early, or later in life, can lead to ocular-related blindness or visual impairment (WHO definition of blindness: visual acuity of 20/200 or worse in the better seeing eye with corrective lenses, or visual field restriction to 20 degrees diameter or less in the better eye. WHO definition of visual impairment: visual acuity of 20/60 or worse in the better seeing eye^1^). In the case of ocular blindness, the rest of the downstream processing structures within the brain appear to remain largely intact despite the loss of visual sensory input early in development. It is important to realize that blindness and visual impairment may also result from acquired damage (e.g., stroke or trauma) occurring at the level of transmission and/or processing structures lying outside of the eye itself. This includes at the level of the geniculate, optic radiations, and primary and associative areas of the visual cortex. Damage to these structures is generally referred to as *neurological visual impairment* (Good, 2007) and is associated with perceptual deficits that are typically more complex than those resulting from damage to the eye (Dutton, 2003; Hoyt, 2003).

In contrast to these two scenarios, there has been a more recent and dramatic rise in the incidence of children born with profound visual impairment not fitting with the typical profile of ocular blindness or visual impairment related to acquired brain injury. In this situation, the term *cortical/cerebral visual impairment* (CVI) was coined to describe damage to visual pathways and structures occurring during early perinatal development (Hoyt, 2003; Good, 2007, 2009). The term “cortical” visual impairment was originally proposed by Whiting et al. (1985) to describe visual dysfunction in pediatric populations of non-ocular cause, and its presumed association with damage to early visual cortical areas. However, as further characterization of this condition progressed, it became evident that CVI was often associated with damage to sites beyond early visual cortex including subcortical structures, white matter pathways, as well as higher-order associative processing areas of the cortex. Thus, the word “cortical” has been viewed as somewhat of a misnomer, and there has been the suggestion that the term “cerebral” would be a more encompassing and appropriate term (Good et al., 1994; Colenbrander, 2010). While the naming convention of CVI (i.e., cortical or cerebral) remains the subject of debate within the education and medical communities, it is important to acknowledge that employing accurate descriptive terms to characterize and localize the site of brain-based injury remains important issue. Two aspects merit consideration in this regard. First, it should be considered that brain injury in CVI may also affect areas beyond those ascribed to visual processing. Indeed, children with CVI often present with other neurological disorders such as cerebral palsy, seizures, or cognitive and developmental delays related to the location of brain damage (Huo et al., 1999; Philip and Dutton, 2014). With this taken into account, it is important to note that the terms cortical and cerebral fail to capture the possibility of more global neurological injury (Good, 2007). Second, injury to the visual system can result in a myriad of visual deficits. While these deficits can occur in isolation or in combination, the current anatomical definition is not sensitive to discriminate between CVI patients based on their apparent perceptual and cognitive dysfunctions. With these limitations in mind, the use of CVI as an umbrella term has emerged, encompassing both clinical and functional characteristics, stating that CVI is defined as a significant deficit in visual function associated with damage to retrochiasmatic visual pathways and cerebral structures in the absence of major ocular disease (or more accurately, the presence of visual deficits that cannot be explained by ocular abnormalities alone) (Dutton, 2003). Within the visual domain, these deficits include decreased visual acuity (ranging from mild to moderate impairment/low vision to profound blindness) as well as visual field impairments (typically in the lower hemi-field) (Good et al., 2001; Kozeis, 2010). Most notably, however, are observed difficulties in higher-order visuospatial processing leading to substantial functional limitations that profoundly impact a child’s learning, mobility, development, independence, and ultimately their quality of life (Fazzi et al., 2004; Boot et al., 2010). This broad spectrum of visual deficits makes the diagnosis of CVI not only more difficult to characterize and quantify, but also raises challenges in terms of developing appropriate and individualized rehabilitative strategies (McKillop and Dutton, 2008; Good, 2009).

There is now, more than ever, a greater need for improved accuracy in diagnosing, assessing, and developing effective education and rehabilitation programs for individuals with CVI. Furthermore, given that visual deficits in CVI are very diverse, their impact upon education and rehabilitative strategies remain much less well understood than in the case of ocular impairment (Baker-Nobles and Rutherford, 1995). This distinction is of utmost importance when considering that educational strategies designed to increase independence and functionality in children with ocular visual impairments are largely ineffective, and perhaps even detrimental, when applied to children with CVI (Groenveld et al., 1990; Farrenkopf et al., 1997). Neuroscience (particularly with regards to advanced neuroimaging techniques) may enable researchers to shed light on to these issues. In particular, it is crucial to uncover the neurophysiological differences between children with CVI and other forms of visual impairment and understand the association between potential risk factors and observed visual deficits. At the same time, this also represents a unique opportunity to rethink traditional interpretations related to what it means to be “visually impaired” (Swift et al., 2008) likewise, explore the developmental potential of the brain.

### Epidemiology and Etiology of CVI

From a public health perspective, CVI represents a pressing issue as it is now the leading cause of congenital visual impairment in children in developed countries including the United States (Good et al., 1994; Huo et al., 1999; Hoyt, 2007; Kong et al., 2012; Philip and Dutton, 2014). In the United Kingdom, CVI is the predominant disorder affecting up to 40–48% of the impaired children younger than 15 years old (Rahi et al., 2003; Rahi, 2007). It is worth noting that while visual impairment worldwide has decreased since early estimates in the 1990s, current evidence suggests that the incidence of CVI is continuing to rise in developed countries. This is due in large part to advancements in the delivery of neonatal intensive care resulting in greater infant survival from neurological damage and complications occurring during pregnancy and perinatal period (Good, 2001; Kozeis, 2010).

Individuals with CVI will also usually present with other coexisting disabilities and neurological disorders. In addition, secondary etiologies associated with CVI are also often present and include seizure, metabolic diseases, and underlying genetic syndromes. These same conditions can also further exacerbate complications and developmental delays accompanying CVI (Flodmark et al., 1990; Good et al., 1994). This makes assessment and evaluation difficult in the setting of concomitant cognitive (including attention), motor (such as cerebral palsy), and language deficits (Good et al., 1994). Finally, it is also crucial to realize that many individuals with CVI (when properly evaluated) are not truly blind *per se* based on their visual acuity alone, though they may ultimately function as a blind individual given inherent difficulties in the processing and interpretation of visual information (Swift et al., 2008).

In characterizing CVI, it is useful to distinguish between children born at term versus those born premature (WHO definition of prematurity is born alive before 37 weeks of pregnancy. Sub-categories of preterm birth based on gestational age include extremely preterm (<28 weeks) and very preterm (28 to <32 weeks)^2^. In infants born term, the most common cause of CVI is perinatal hypoxia-ischemia encephalopathy (HIE) (Huo et al., 1999; Fazzi et al., 2007; Khetpal and Donahue, 2007). The sequelae of HIE are dependent not only on the severity and duration of the hypoxic event, but also the gestational age (Hoyt, 2003). Specifically, regional differences in vascular perfusion (e.g., “watershed” zones; Baburamani et al., 2012) and the higher metabolic demand of the near term fetus (Bennet et al., 1999) alter the susceptibility of different brain locations to hypoxic-ischemic damage as the baby matures (Van den Broeck et al., 2008). In HIE, the areas that are most commonly damaged include deep gray matter, hippocampus, brainstem, and thalamic regions (Swarte et al., 2009). In contrast, premature infants will often present with periventricular leukomalacia (PVL) as the most common form of brain injury (Van den Broeck et al., 2008). This is associated with hemorrhagic necrosis in the periventricular white matter just dorsal and lateral to the external angle of the lateral ventricles (Volpe, 1998). The main factors commonly associated with PVL are an underdeveloped vasculature of the surrounding white matter, as well as impairment of the regulation of cerebral blood flow; both of which can predispose white matter to ischemic injury. Crucially, as the tracts of the optical radiations and of higher order visual functions travel within the periventricular white matter, PVL is often associated with impaired visual processing (Good, 2001; Dutton, 2003; Hoyt, 2007).

In both preterm and term infants, a common consequence is cell death (i.e., necrosis) of myelinated and pre-myelinated fibers obstructing the normal development of white matter pathways that communicate between sensory and motor areas of the brain. As a result, children with CVI will often exhibit motor and cognitive impairments associated with cerebral palsy (Lim et al., 2005).

While perinatal hypoxia remains the most common cause of CVI, other possibilities include traumatic brain injury (TBI; 10% of the cases in one reported study; (Khetpal and Donahue, 2007) as well as infectious etiologies (e.g., meningitis and encephalitis) leading to inflammatory-mediated white matter injury (Huo et al., 1999). Finally, seizure disorder is also a significant cause of CVI (estimated at 10% of cases) and also represents the most common associated neurological abnormality [reported in nearly 50% of CVI cases (Huo et al., 1999)].

### Compensatory Behaviors and Crossmodal Plasticity in the Setting of Visual Impairment

It is clear that in a world which heavily relies on sight, blind individuals have to make striking adjustments in order to remain functionally independent. Accumulating evidence suggests that individuals with ocular blindness (particularly, when blind from birth or very early in life) demonstrate comparable, and in some cases even superior, behavioral skills in the tactile and auditory domains as compared to their sighted counterparts (e.g., Lessard et al., 1998; Van Boven et al., 2000; Amedi et al., 2003; Gougoux et al., 2004; Wong et al., 2011; for review, see Merabet and Pascual-Leone, 2010). This has led to the suggestion that compensatory behaviors may be intimately related to underlying changes in the overall structural and functional organization of the brain resulting from profound vision loss (Voss et al., 2014). Interestingly, it has been shown that this reorganization implicates areas of the brain responsible for the processing of intact senses such as touch, hearing, and smell (e.g., Sterr et al., 1998; Hamilton et al., 2004; Rombaux et al., 2010) as well as the crossmodal reorganization of areas of the brain normally associated with the processing of visual information. Regarding the latter, numerous neuroimaging studies (predominately functional magnetic resonance imaging, or fMRI) have demonstrated that blind individuals show robust activation within occipital cortical areas while performing a variety of non-visual tasks [e.g., Braille reading (Sadato et al., 1996), sound localization (Gougoux et al., 2005; Voss et al., 2008; Collignon et al., 2011), and odor perception (Kupers and Ptito, 2014), as well as higher order cognitive tasks including language processing (Burton et al., 2002; Bedny et al., 2011; Striem-Amit et al., 2012) and verbal memory recall (Amedi et al., 2003; Raz et al., 2005). In summary, there appears to be mounting evidence supporting the view that the brain undergoes dramatic structural and functional changes in response to ocular blindness.

While the vast majority of scientific research investigating structural and functional brain changes resulting from profound visual deprivation has been carried out within the context of ocular causes of blindness, comparatively little research has been conducted investigating the developmental repercussions and neuroplastic compensatory mechanisms in CVI. As mentioned earlier, this is despite the high prevalence of this condition and its potential detrimental consequences on visual function and development. Indeed, often in the absence of ocular signs or pathology (combined with a lack of awareness of this condition by health care providers) many children with CVI are misdiagnosed. As such, often their visual difficulties are mistakenly attributed to a behavioral and psychological disorder (Swift et al., 2008). For those children that are accurately diagnosed, there remains the issue of the aforementioned observation that education and rehabilitation strategies developed for people with ocular blindness are not effective in the case of CVI (Groenveld et al., 1990; Farrenkopf et al., 1997). Put another way, how is it that two individuals with a comparable level of visual impairment (as characterized by measured visual acuity) could respond differently to the same training strategy? Could this be related to underlying structural and functional changes within the brain that differ between ocular compared to cerebral causes of visual impairment? In other words, does the brain develop and adapt differently in the setting of damage to visual cerebral structures compared to damage to the eye? Uncovering these differences would be of utmost importance not only in terms of developing appropriate education and rehabilitative strategies, but also to help better characterize the underlying physiology of CVI.

### CVI: A Disorder of Brain Connectivity?

As mentioned previously, children and adolescents with CVI often present with marked impairments in visual function including decreased visual acuity and visual field deficits impairments (Good, 2001; Kozeis, 2010). However, most striking are their deficits in the processing of higher-order and complex visual information (Fazzi et al., 2004; Boot et al., 2010). This includes cognitive and visuomotor processing difficulties related to object and spatial processing tasks (i.e., identifying common objects such as faces and locating them in space). Often, individuals with CVI will exhibit difficulties in locating a target object in a crowded or complex visual scene. For example, a child with CVI will often report not being able to identify a parent in a crowd or locate their favorite toy in a box filled with other toys (Dutton, 2003). They often will report troubles with finding their way around as well as perceiving complex moving scenes including interpreting biological motion (Braddick et al., 2003).

In describing visual processing, the concept of a two-stream hypothesis (i.e., dorsal/spatial processing and ventral/object processing) has often been purported in order to explain the division of labor as to how certain attributes are analyzed within a visual scene (Mishkin and Ungerleider, 1982). Given the nature of the visual dysfunctions observed in CVI, certain investigators have proposed that CVI may be a condition best characterized as a dorsal stream “dysfunction” or “vulnerability”; consistent with an impairment in the functioning of the dorsal/spatial visual processing pathway (i.e., connecting the occipital to parietal cortices and terminating in frontal areas) (Braddick et al., 2003; Dutton, 2009; Taylor et al., 2009). Certainly, this dichotomy represents a practical and useful conceptual framework to characterize observed deficits in CVI (Dutton, 2003). However, it is worth noting that neurophysiological support for this concept remains lacking despite strong psychophysical evidence. Furthermore, spatial processing deficits are not universal in CVI, nor do they occur in isolation from other deficits that can be characterized as non-spatial in nature. Indeed, many individuals with CVI also exhibit a broad spectrum of visual dysfunctions including object identification such as recognizing faces and shapes (Good et al., 1994; Porro et al., 1998; Fazzi et al., 2004, 2007, 2009). The nature of these perceptual impairments remains unclear in that is unknown whether these deficits represent true “agnosias” or rather are related to other cognitive issue such as imagery, language, and memory (see Fazzi et al., 2009, for further discussion). According to the two stream hypothesis, impairments in object identification (e.g., faces, toys, and other objects) would be suggestive of damage along the ventral visual processing pathway (i.e., connecting the occipital and temporal cortices) which must also be explained from a neuroanatomical and neurophysiological point of view. Lastly, concomitant oculomotor and attentional issues are also often present in individuals with CVI, which are not typically ascribed to the two stream visual processing model, but rather, may implicate different pathways and structures entirely. Therefore, while damage along key visual processing streams may be associated with observed perceptual deficits, the underlying maldevelopment of the brain in CVI appears to be more extensive and complex than previously assumed.

Indeed, clinical editorials have highlighted that the relationship between observed clinical manifestations and the extent of brain damage in CVI is complex and not yet fully understood (Guzzetta et al., 2010). Additionally, attention has been called to the value of advanced neuroimaging techniques in helping to better understand the relationship between brain maldevelopment and visual impairment in CVI (Good, 2001; Edmond and Foroozan, 2006; Murakami et al., 2008). With this in mind, early studies have attempted to associate visual impairments in CVI with alterations in brain structure using standard clinical neuroimaging modalities (predominantly magnetic resonance imaging, or MRI). For example, Serdaroglu et al. (2004) reported that the degree of gross cerebral morphological changes (and in particular, the severity of PVL) was correlated with neurodevelopmental outcomes. For example, children with low severity of PVL had minor motor problems or mild to normal functional outcomes, whereas the presence of cortical atrophy and thinning of the corpus callosum were associated with more developmental delays (Serdaroglu et al., 2004). While standard clinical neuroimaging techniques like structural MRI and computerized tomography (CT) can help characterize gross changes in cerebral structure, the underlying microarchitecture of white matter pathways cannot be ascertained. In the case of CVI, this is particularly relevant in terms of the need to characterize the relationship of specific types of visual impairments with key processing pathways such as the optic radiations and dorsal and ventral processing streams. There are advanced neuroimaging techniques that enable the examination of brain structure and anatomical pathways in a more detailed manner that may help further our understanding of the neuroanatomical basis of CVI. In particular, diffusion based imaging studies (such as diffusion tensor imaging, or DTI) combined with white matter tractography analysis can be used to reveal the organization of white matter pathways and thereby reveal how the brain is inter-connected. Briefly, by tracking the diffusion movement of water molecules in the brain, the overall organization of white matter connectivity can be inferred (Jones, 2008; Ffytche et al., 2010). Furthermore, it is possible to employ tractography techniques that allow for the “virtual dissection” of the brain so that pathways to be reconstructed and individually examined (Catani and Thiebaut de Schotten, 2008). For example, it is the neuroanatomical correlate of the dorsal/spatial stream has been identified as the superior longitudinal fasciculus (SLF) (Catani and Thiebaut de Schotten, 2008). In contrast, the inferior longitudinal fasciculus (ILF) represents the neuroanatomical correlate of the ventral visual processing stream (Catani and Thiebaut de Schotten, 2008). It is also important to identify a third pathway (though previous reports associate this pathway as part of the dorsal pathway; see Schmahmann et al., 2007; the inferior fronto-occipital fasciculus (IFOF) which appears to the be associated with visual attention and eye movements given its strong connections between occipital and frontal areas (Ffytche et al., 2010). Thus, using advanced diffusion based imaging techniques and white matter tract reconstruction, there is the unique opportunity to explore the association between visual perceptual deficits and the structural integrity of the visual pathways that support normal visual function and development.

In the specific case of CVI, previous studies using diffusion based MRI have identified marked alterations in white matter structure and further suggest that there is an association between the maldevelopment of key visual pathways and the visual dysfunctions observed in this condition. In one recent study by Lennartsson et al. (2014), diffusion weighted MRI was carried out in a group of individuals with white matter damage predominantly in the superior posterior periventricular white matter and with documented visual dysfunction. Specifically, it was found that early injury to the optic radiations was associated with characteristic patterns of visual field deficits. Interestingly, in a review study by Guzzetta et al. (2013), this group reported that many individuals diagnosed with CVI and with early periventricular damage to the optic radiations often showed normal development of visual field function. In their review, the authors suggested that the preservation of visual field function they reported may be the result of compensatory neuroplastic reorganization; an important observation that needs further and careful study as this may have important rehabilitative implications (Guzzetta et al., 2013).

**FIGURE 1:**
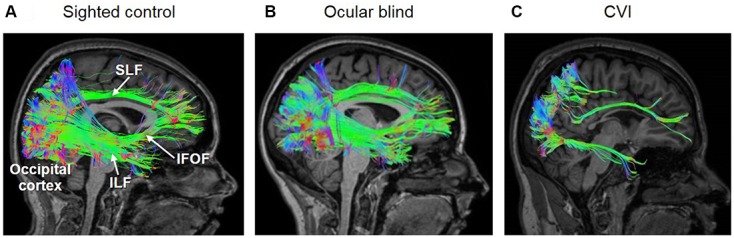
**White matter reconstructions (shown in sagittal view) of three main pathways involved in the processing of visual information, namely the superior longitudinal fasciculus (SLF; the neuroanatomical correlate of the dorsal visual processing stream), inferior longitudinal fasciculus (ILF; the ventral visual processing stream), and inferior fronto-occipital fasciculus (IFOF; mediating visual attention and orienting).** Diffusion data was acquired using 64 direction high angular resolution diffusion tensor imaging (HARDI). The pathways were reconstructed in DSI-Studio (Yeh et al., 2010, 2013) using individual QA termination thresholds and a termination angle of 45 degrees. The three white matter pathways are reconstructed in **(A)** a normally sighted/developed control, **(B)** early ocular blind, and **(C)** and CVI (with associated periventricular leukomalacia) individuals. Note that all three pathways (ILF, SLF, and IFOF) are fully reconstructed in both the control and early ocular blind individuals. In contrast, the SLF and ILF are sparser, and the IFOF was could not to be reconstructed in the individual with CVI. These differences in the structural integrity along these major white matter pathways may be related to observed cognitive visual dysfunctions in CVI [Figure adapted from Bauer et al. (2014b) and Hirsch et al. (2015)].

A number of recent studies have investigated the individual pathways implicated in the processing of visual information between cortical areas of the brain with the aim of establishing a possible association between the structural integrity of these pathways and the visual dysfunction in CVI. In one study, the integrity of the SLF (measured by fractional anisotropy, or FA) was examined using DTI in association with impairments in object identification observed in a cohort of individuals with CVI. Specifically, it was shown that the structural integrity of the ILF was significantly decreased in CVI compared to normally developed controls (Ortibus et al., 2011). Given the role supported by the ILF, SLF, and IFOF in various aspects of visual processing (object, spatial, as well as visually guided attention and eye movement control, respectively), it seems plausible that the visual impairments observed in CVI could be associated with specific structural changes in white matter connectivity at the individual level. Indeed, in a recent report by two of the coauthors of this review (Bauer and Merabet), individuals with CVI were shown to have dramatic reductions in the volume and number of fibers of the ILF, SLF, and IFOF (Bauer et al., 2014b). To characterize potential differences in white matter connectivity and integrity, these investigators employed high angular resolution diffusion imaging (HARDI) rather than DTI as was previously done in aforementioned studies. While both DTI and HARDI techniques provide information regarding the degree of water diffusivity in the brain in order to derive local axonal fiber orientation, it is becoming increasingly established that HARDI is superior in its ability to delineate the organization of crossing fibers, and ultimately the overall microarchitecture of the brain (Tuch, 2004; Jones, 2008; Tournier et al., 2011). Interestingly, preliminary results from Bauer et al. (2014a) showed that these dramatic reductions in all three fasciculi were not observed in ocular blind subjects as compared to normally sighted/developed controls (**Figure 1**). These early findings using diffusion based MRI techniques and whiter matter tractography reconstruction appear to support the notion that the brains of individuals with CVI show dramatic differences compared to normally developed brains as well as the case of ocular blind individuals. Finally, in a recent case study using a combined HARDI and fMRI approach, Merabet et al. (2016) demonstrated that in a CVI subject with a clinically documented inferior visual field deficit (assessed by formal perimetric testing), there was a structural-functional correspondence between the location and extent of the visual field deficit, damage to superior branches of the optic radiations (characterized by HARDI), and reduced retinotopic activation of early visual cortical areas associated with the representation of the inferior visual field (as indexed by fMRI). This correspondence is in accordance to the known anatomical and functional organization of visual pathways and geniculo-cortical representation of visual field space (Wandell, 1999) and demonstrates the advantage of a combining a clinical and multimodal neuroimaging approach to help characterize the underlying neurophysiology of visual deficits in CVI.

Taken together, these studies suggest that CVI may be associated with a more generalized vulnerability implicating numerous key pathways supporting the developing visual system. Furthermore, neuroplastic changes within the developing brain (such as the “re-wiring” of key geniculo-cortical or cortico-cortical connections) may support the sparing of visual function in certain individuals with CVI. It is important that future work be focused on correlating the degree of structural impairment of individual processing pathways with a broad range of measured outcomes of visual processing deficits at the individual level.

**FIGURE 2:**
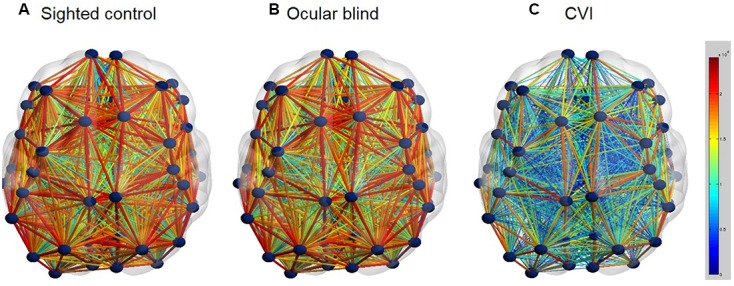
**Whole brain structural connectivity (ball and stick models; axial view) for the group averages of normally developed sighted controls (A)**, ocular blind **(B)**, CVI individuals **(C)**. White matter connectivity across the entire cortex was assessed using HARDI tractography. The number of reconstructed fibers between each of the 68 cortical regions (parcellated using the Desikan atlas; Desikan et al., 2006)) was used as a proxy for connection strength. Similar to the data shown in **Figure 1**, connections between each region were reconstructed in DSI-Studio. Once whole brain connectivity matrices were acquired for all subjects, they were averaged within subject groups and visualizations were rendered using BrainNetViewer (Xia et al., 2013). Each brain region is represented by a dark blue sphere, while the connection strength (i.e., number of reconstructed connections) between each region is represented by the color and diameter of the lines. Thus, a thick red line characterizes strong connections with abundant white matter fibers, while a thin blue line characterizes weak connections with minimal white matter fibers. Note the striking reduction in global structural connectivity that occurs in CVI, compared to both the control and ocular blind individuals [Figure adapted from Bauer et al. (2014a)].

While the extent and integrity of individual visual processing pathways can be investigated using diffusion based imaging techniques, it is also possible to use the same data to explore whole brain networks of connectivity of the entire brain (Sporns et al., 2005). Specifically, comparing whole brain network connectivity between CVI and ocular blind individuals may further provide insight into developmental differences between these two groups. In a preliminary analysis, comparison of whole brain connectivity networks was carried out based on white matter connectivity derived from HARDI (**Figure 2**). In this analysis, it was found that whole brain connectivity was very similar when comparing ocular blind and normally sighted controls (Bauer et al., 2014a). As with the case of examining the dorsal and ventral pathways independently, robust network connectivity throughout the brain was evident in individuals with ocular blindness and may further be related to the compensatory behaviors observed in this group (Merabet and Pascual-Leone, 2010). In contrast, whole brain connectivity appeared to be markedly reduced in individuals with CVI as compared to ocular blind and normally sighted controls (Bauer et al., 2014a). While further confirmation of these results are ongoing, they do suggest that a global impairment in overall brain connectivity may be associated with observed cognitive visual dysfunctions as well as other associated sensorimotor and cognitive delays in CVI. This level of whole brain analysis may also provide hints as to why education and rehabilitative strategies designed for individuals with ocular blindness may not be effective in the case of CVI.

The use of advanced neuroimaging modalities like diffusion based imaging in CVI are still in the early stages. However, it is becoming clearer that much can be learned regarding the underlying neurophysiology of this condition beyond what can be ascertained by standard structural imaging alone. This may help uncover associated links between underlying brain connectivity and cognitive visual dysfunction in CVI and provide clues as to how to develop novel education and rehabilitation strategies for individuals living with blindness and profound visual impairment. Equally evident is that the diagnosis of visual impairment/blindness based on singular criteria such as visual acuity fails to accurately characterize the true overall functioning, as well as potential, of an individual. In this regard, better characterization of visual deficits using a more even-handed and comprehensive testing battery will be important. Finally, it will also be important to associate, as well as disentangle, the contributory effects of potential risk factors associated with CVI and further establish their relationship to overall prognosis. For example, while there may be a suspected link between CVI and certain developmental disorders such as autism spectrum disorder (ASD), clear epidemiological evidence is still lacking (note that there is mounting evidence establishing low birth weight, prematurity, and neonatal encephalopathy as important risk factors for ASD and other cognitive delays; (Badawi et al., 2006; Schendel and Bhasin, 2008; Schieve et al., 2014). Indeed, the neurobiological impact of prematurity on the brain is likely to be extensive as well as variable. Thus the ability to parse out observed perceptual, cognitive, and motor deficits as a function of underlying developmental impairments remains crucial.

## Conclusion

Studies using advanced neuroimaging techniques have contributed greatly to our understanding of how the brain adapts to the loss of sight and have helped uncover the neuroplastic mechanisms that relate to compensatory behaviors. While considerable knowledge has been gained in the study of individuals living with ocular visual impairment, a similar concerted effort is needed to gain important insight regarding the visual dysfunctions of children and adolescents with CVI. This may help to better understand the interrelationship between specific developmental deficits, underlying brain anatomy and function, and compensatory behavioral adaptations. In the end, understanding the conditions that promote neuroplasticity within the brain, both in the setting of ocular and cortical/cerebral blindness, will be crucial to help to maximize the learning, development, and well-being of these individuals.

## Author Contributions

Study concept and design: MBM and LM. Study of CVI vs. Visual Impairment: MBM, AL-M, and MM. Epidemiology of CVI: AS-L, JM-H, and CB. Etiology of CVI: MBM, AS-L, and JM-H. Relationship between Brain Damage and CVI: CMB and LM. Study of neuroimaging techniques: CMB, LM, and CB. Critical revision of the manuscript: all authors.

## Conflict of Interest Statement

The authors declare that the research was conducted in the absence of any commercial or financial relationships that could be construed as a potential conflict of interest.

## Footnotes

1 http://www.who.int/mediacentre/factsheets/fs282/en/

2 http://www.who.int/mediacentre/factsheets/fs363/en/
